# Barriers and facilitators to implementation of peer support after miscarriage: a systematic review using thematic synthesis methods

**DOI:** 10.1136/bmjopen-2025-113671

**Published:** 2026-06-03

**Authors:** Leanne Burton, Juanita Charles, Mary Gemma Cherry, Rhiannon Corcoran, Shaima Hassan, Ruaraidh Hill, Sarah Little, Michelle Maden, Helen Mulholland, Elizabeth Perkins, Pauline Slade, Selina Wallis, Paul Marshall

**Affiliations:** 1 Department of Primary Care & Mental Health, University of Liverpool, Liverpool, UK; 2 The Miscarriage Association, Wakefield, UK; 3 Clinical Health Psychology, University of Liverpool, Liverpool, UK; 4 Department of Health Data Science, University of Liverpool, Liverpool, UK; 5 Liverpool Reviews and Implementation Group, University of Liverpool, Liverpool, UK; 6 Independent Researcher (Public Advisor), Cheshire, UK

**Keywords:** MENTAL HEALTH, Systematic Review, Pregnancy, Peer Group

## Abstract

**Abstract:**

**Objectives:**

Miscarriage, defined in the UK as loss of pregnancy prior to 24 weeks gestation, can have long-term psychological implications. Clinical guidelines for perinatal bereavement care do not provide guidance on how best to support the mental health of women, and their partners, after miscarriage. Peer support (support from those who share common characteristics) is often sought, but there is little understanding of its access and use. We conducted a systematic review to understand the barriers to and facilitators of the implementation of peer support to improve mental health outcomes for parents after miscarriage.

**Design:**

Systematic review and thematic synthesis.

**Data sources:**

A comprehensive systematic search across nine databases (MEDLINE, CINAHL, APA PsycINFO, Web of Science (all databases), EMBASE, CENTRAL, LENS.org, British Nursing Index and Health Management Information Consortium) was conducted in June 2025. Grey literature was identified through website searching, contact with topic experts and a national Call for Evidence.

**Eligibility criteria:**

Qualitative and mixed-methods studies exploring motivations, experiences and preferences for peer support after miscarriage were included.

**Data extraction and synthesis:**

Two independent reviewers used standardised methods to search, screen, extract and code included studies. Suitable studies were evaluated using the Critical Appraisal Skills Programme Qualitative Research Checklist. Findings were extracted and subjected to a thematic synthesis.

**Results:**

Across nine studies included in the review, three overarching themes were developed, with seven subthemes, capturing both barriers and facilitators. ‘Engaging in relational recognition’ reflects the validation and connection that arise through experiential resonance, often heightened by the context of exclusion from broader social or clinical support. ‘Mechanisms of Communality’ describe how communality is enacted through dynamic peer interactions, including modelling and facilitating grief, benchmarking physical change and mattering through reciprocity, highlighting mutual support and shared coping. ‘Dynamics of Access’ consider factors which shape engagement, including changing needs of individuals across time and modalities of support and their effects.

**Conclusions:**

These findings form the first synthesis of peer support after miscarriage and bring a nuanced service user perspective of barriers and facilitators by examining evidence from diverse studies. Peer support after miscarriage was seen to be a dynamic, relational process shaped by shared experience, mutual exchange and context-specific factors. Findings underscore key policy and practice considerations, including the use of trauma-informed, loss-sensitive approaches and consideration of intersectionality, that should be reflected when offering peer support services, with and for, those who have experienced miscarriage.

**PROSPERO registration number:**

CRD42024518248.

**STRENGTHS AND LIMITATIONS OF THIS STUDY**

- Study design that incorporated a thorough literature search, including grey literature searching and a Call for Evidence, to allow for a detailed investigation of current evidence.
- A thematic synthesis approach was used, a key aim of which is to help inform future service design, policy and clinical practice.
- Inclusion of studies from multiple countries allowed identification of common barriers and facilitators, as well as how these issues vary within and between contexts.
- This review is limited by the heterogeneity and quality of included studies and restrictions to English language, potentially restricting the transferability of review findings and the evidence base.

## Background

 Miscarriage, defined in the UK as loss of pregnancy prior to 24 weeks gestation, affects one in five women in their lifetime.^1^ Miscarriage is a bereavement and, naturally, is associated with a range of psychological responses including grief, anxiety and trauma. Its experience can be very difficult for both women and their partners.^2^,^5^ Anxiety is the most common and persistent psychological disorder following miscarriage,^4^ with evidence of depression and post-traumatic stress disorder also experienced in both the short and long-term.^2 6^ The psychological effects of miscarriage can have implications for family functioning, relationships, future pregnancies, employment opportunities and experiences, and financial expenses, as well as additional healthcare service utilisation.^7^,^10^

The National Health Service clinical guidelines for perinatal bereavement care do not provide guidance on how best to support the mental health of women, and their partners, after miscarriage. The UK’s 2023 Pregnancy Loss Review highlights current insufficiencies in mental health support after miscarriage, and makes recommendations that mental health support is offered, where necessary, to both parents.^11^ Previous studies highlight a need for emotional, informational and practical support in the period following miscarriage.^12^,^14^ However, research has shown that obtaining appropriate support can be challenging.^15 16^ Women often encounter silence, stigma and inadequate care from both personal networks and healthcare providers after experiencing miscarriage.^17 18^

In the past 20 years, peer support has become increasingly popular, particularly in the mental health landscape.^19^ Peer support is a powerful connection in human behaviour^20^ and has received a strong emphasis in the commissioning plans of the recently formed Maternal Mental Health Services in the UK.^21^,^23^ Peer support is defined as *“the provision of emotional, appraisal and informational assistance by a created social network member who possesses experiential knowledge of a specific behaviour or stressor and similar characteristics as the target population”* (p.329).^24^ Peer support connects an individual to another person (a peer) who has been through a similar experience, to provide support, hope and encouragement.^25 26^ It is viewed as different from, and complementary to, professional healthcare, hypothesised to work by building social relationships that have reciprocal benefits to both health and well-being such as improved self-esteem, self-efficacy and hopefulness.^26^,^29^

While peer support is not conceptualised as a mental health intervention, the hope and encouragement that peer support brings may have positive psychological impacts. Studies of the effectiveness of peer support for mental health have mixed findings.^27 30 31^ Cooper *et al*’s umbrella systematic review and meta-analysis found that peer support may reduce perinatal depression and improve recovery outcomes, self-efficacy and stigma-related outcomes.^27^

At service provider level, research suggests peer supporters require additional training, support and supervision, as well as clarity around roles and their integration into the wider multidisciplinary team.^27^ A study of telephone peer supporters following miscarriage conducted in Australia found that while volunteer providers felt confident and prepared, key challenges included workload, sustainability and ongoing training and support.^32^ While some evidence exists from the provider perspective, it is important to consider the unique, unmet and context-specific challenges that those who experience miscarriage face. Evidence suggests that traditional mental health support often fails to meet the emotional, informational and esteem needs following miscarriage.^32 33^ To date, a review of the evidence of service users’ experiences of peer support after miscarriage has yet to emerge.

The objective of this systematic review was to address one of two research questions outlined in the PROSPERO registration: “*What are the barriers to and facilitators of implementation of peer support to improve mental health outcomes for parents after miscarriage?”*. The other registered question will be addressed separately and is therefore not considered in the present review. Using qualitative and mixed-methods studies, the review will explore motivations, experiences and preferences for support services, as well as barriers to participation. A reflection on the findings will be useful to inform future research and for practitioners designing and implementing peer support after miscarriage.

## Methods

The review followed the Preferred Reporting Items for Systematic Reviews and Meta-Analyses (PRISMA) guidelines. A review protocol was preregistered with the International Prospective Register of Systematic Reviews (PROSPERO; registration number: CRD42024518248).

### Literature search and study selection

Eligible studies were identified through a systematic search of nine electronic databases: MEDLINE, CINAHL, PsycINFO, Web of Science (all databases), EMBASE, CENTRAL, LENS.org, British Nursing Index and Health Management Information Consortium, grey literature searching and a Call for Evidence.^34^ Search terms were discussed and agreed within the research team and study public advisory group (see online supplemental appendix 1). The initial search was conducted in July 2024 and updated in June 2025. Reference lists of all included studies were screened, and forward citation searching was conducted using Google Scholar.

Following de-duplication, titles and abstracts of all identified citations were imported into Rayyan.ai (web-based platform for managing systematic reviews^35^) for independent screening by two reviewers (LB and EW). Disagreements were resolved through discussion and consensus, with a third reviewer (PM) consulted when necessary.

### Eligibility criteria

Eligibility criteria are outlined below (table 1):

**Table 1:** Eligibility criteria for studies considered for inclusion in the review

	Inclusion	Exclusion
Participants	Parent(s) (ie, women and birthing people or their partners) with experience of miscarriage (loss of pregnancy up to 24 weeks gestation) who accessed peer support.	Studies conducted with a population who had experienced later pregnancy loss, stillbirth or infant loss, where it was not possible to separate out miscarriage-related data (<24 weeks gestation) Peer support in other population groups (eg, grandparents)
Interventions	Peer support, defined as the existence of a community of common interest where people gather (in person or virtually by telephone or computer) to share experiences, ask questions, provide emotional support and gain self-help. Peer support which uses a formal or professional facilitator, provided the role of the facilitator is to manage group interpersonal processes rather than provide counselling or any other psychoeducation.	Services which are not peer support (ie, any other form of psychosocial or psychoeducational support), including where offered as an adjunct to peer support. Peer support offered directly by peer’s family members (ie, partners, parents).
Comparator	No comparator	
Outcomes	Any experiential research studies with an evaluative component, that is, service users’ views of participating in peer support.	Studies describing the use of peer support in practice with no evidence of outcome or process evaluation.
Design	Study designs with a qualitative or mixed-method evaluative component.	Conference abstracts, protocols, reviews and quantitative studies.

In this review, we define ‘peers’ as people who are not trained medically or as a specialist mental health professional but are like the target population because they have similar or relevant health experience. Peers can be those who share common characteristics with a specific individual or group.^24^ Studies with mixed samples (eg, qualitative studies reporting experiences of general pregnancy loss support groups) were included where miscarriage-specific data could be disaggregated. As support groups commonly include individuals with different types and gestations of pregnancy loss (eg, early and late miscarriage and stillbirth), the review team reached consensus that ‘pregnancy loss’ constituted an acceptable shared lived experience for peer support, consistent with the definition of peers as individuals with similar or relevant health experience.

The definition of peer support was kept broad, encompassing diverse types and formats. Studies reporting on online communities, including online forums, email communities and social media-based support groups were included. Online communities were defined as *“virtual social space(s) where people come together to get and give information or support, to learn or to find company*”.^36^ Online groups, both ‘open’ (ie, anyone on the platform can see who is in the group, what posts are shared and can join without needing approval) and closed (ie, only invited members, or members who are approved to join by a moderator, can see the content posted within), were included if they were being used with the specific purpose of offering miscarriage support (ie, share experiences, ask questions, provide support and gain self-help). Studies reporting social media use more broadly, to discuss miscarriage experience and seek support, were excluded from the review.

### Data extraction

Data were extracted and tabulated in Microsoft Excel, including author details, publication year, country, study aims, study design, sample size, peer support ‘intervention’ description (using the Template for Intervention Description and Replication (TIDieR) Checklist),^37^ and the main data, including first order (participant quotes) and second order (author interpretations), salient to barriers and facilitators of implementation. Data were extracted independently by one reviewer (EW) and checked by a second reviewer (LB). Disagreements were resolved through discussion and consensus.

### Quality appraisal

The Critical Appraisal Skills Programme (CASP) tool for qualitative research was used to assess the quality of included studies.^38^ Studies were assessed for quality on 10 items: aims, methods, design, recruitment, method of data collection, researcher–participant relationship consideration, ethics, rigour of data analysis, clarity of findings and value. Each study was rated against each item using a scoring system of 1=criteria met, 0.5=criteria partially met and 0=criteria not met. Studies were given a summed score (maximum of 10) and subsequently rated as high, moderate or low quality.^39^ Two reviewers (LB and EW) conducted quality appraisal of all included studies, and any disagreements resolved through discussion and consensus.

### Data analysis

Thematic synthesis was employed to analyse findings from multiple qualitative studies. Thomas and Harden’s thematic synthesis guidelines were used to direct the analysis process.^40^ First, data from each of the included studies was free coded. Two researchers then aggregated similar codes into descriptive themes using labels, staying as close to the original data as possible. Lastly, the review team worked together to generate new analytical themes by exploring similarities, differences and patterns in the descriptive themes and interpreting these in relation to the review aim. Theme development was discussed regularly at review team meetings. Assimilated themes were used to produce a suggestive list of barriers and facilitators to peer support after miscarriage. One reviewer (LB) independently analysed all data, with a second reviewer (PM) analysing 30% of the data and supporting theme development. Data analysis was managed with NVivo V.15 software.

### Reflexive statement

Being transparent about one’s position is particularly important when conducting a thematic synthesis.^41 42^ The first author’s experiential knowledge provided valuable contextual understanding and heightened sensitivity to issues surrounding peer support after miscarriage. At the same time, the proximity to the topic required ongoing reflexive awareness of the potential influence of personal perspectives on interpretation of the data. A reflexive log alongside regular team discussions allowed for critical examination of emergent themes from multiple disciplinary and gendered perspectives. This improved transparency of the review, supporting the balance between experiential insight and analytical objectivity.

## Results

**Figure 1:**
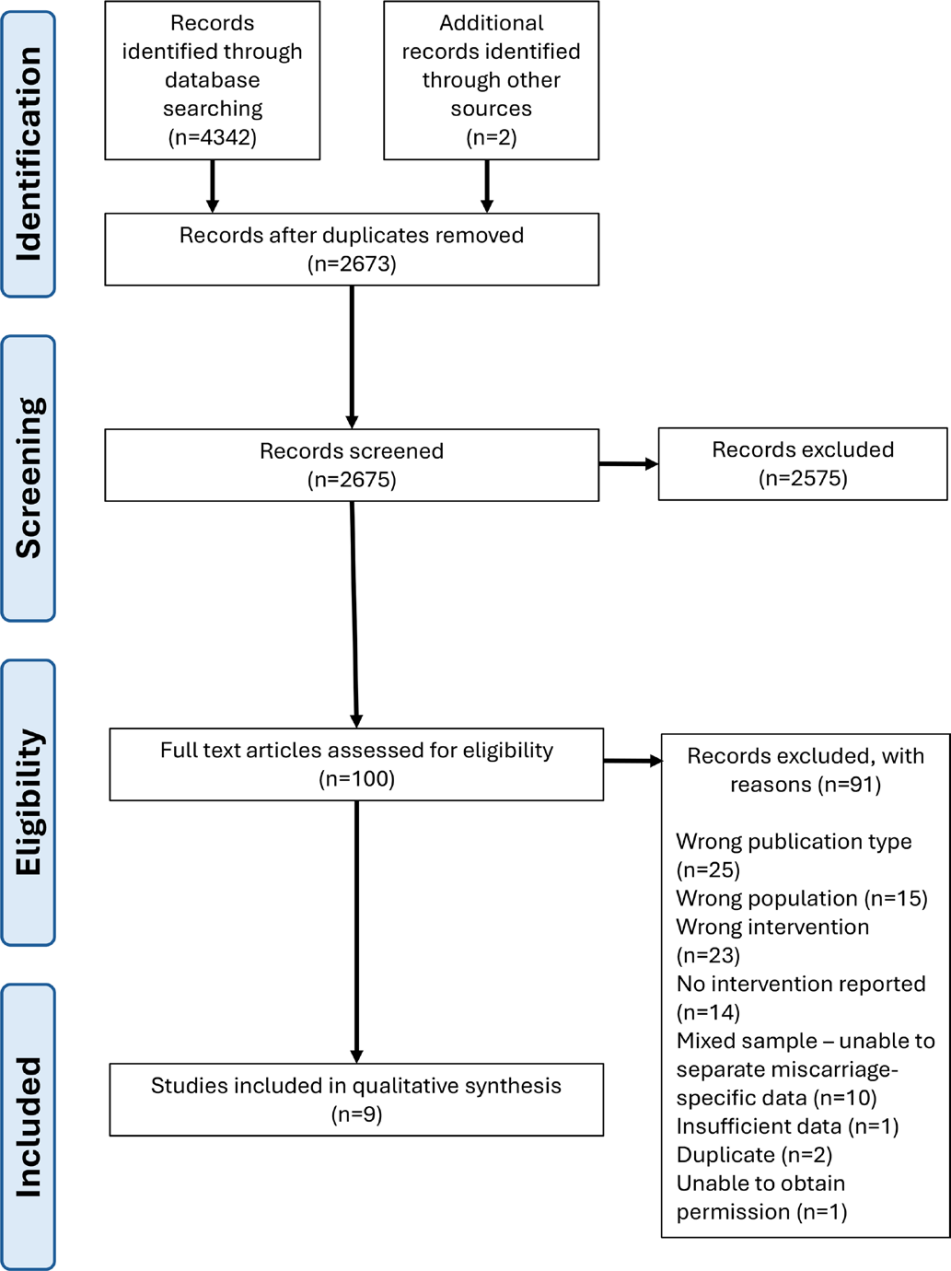
PRISMA flow diagram of study selection. Flow diagram illustrating the process of study identification, screening, eligibility assessment and inclusion in the systematic review, conducted in line with PRISMA guidelines. PRISMA, Preferred Reporting Items for Systematic Reviews and Meta-Analyses.

The combined database search retrieved 4342 records, leaving 2673 after duplicates were removed. Grey literature searches identified two other sources; therefore, the title and abstract of 2675 records were screened. Of these, 100 potentially relevant full-text papers were retrieved and screened, resulting in 9 studies eligible for inclusion (see figure 1 PRISMA flow diagram).

### Characteristics of included studies

*Appendix 2* summarises the characteristics of included studies. The nine included studies were published between 2002 and 2024, with six published within the last 2 years. Most studies were conducted in the USA^43^,^48^; the remainder took place in Japan,^49^ Australia^50^ and the UK.^51^ It is important to note that there is inconsistency in the definition of miscarriage, with definitions varying by country and by upper gestational age and pregnancy viability. This review used an operational definition of miscarriage as pregnancy loss prior to 24 weeks gestation, aligning with that outlined by the UK’s Royal College of Obstetrics and Gynaecologists.^52^

Reporting of participant numbers was inconsistent across studies. Where studies reported the sample size, the total number of participants who had experienced miscarriage across the studies was at least 63 (54 females, 8 males (partners) and 1 missing data). Three papers which analysed archival online data were unable to report on participant numbers due to the nature of the dataset.^44 48 50^ Reporting of participant age, ethnicity and religion varied between studies; see online supplemental appendix 2.

While all nine studies met inclusion criteria, they varied in their provision of demographic information about participant loss experiences. It was not possible to determine specific gestation of losses in seven of the studies as it was not clearly defined (ie, late first trimester). Two studies, comprising 24 participants, reported loss gestation more clearly with losses occurring from 7 to 23 weeks gestation.^49 51^ Five studies did not report on whether miscarriage was experienced as a singular event or had previously occurred in participants.^43^,^4549 50^ One paper specifically recruited participants who had experienced recurrent miscarriage (defined as three or more first trimester miscarriages).^48 53^ Three other papers, comprising 22 participants, reported 14 participants having had a singular miscarriage, while the remaining 8 had experienced recurrent miscarriage.^46 47 51^

In regard to data collection, one study adopted a mixed methodological approach,^49^ with the remaining studies being exclusively qualitative. Five studies used semistructured interviews as part of the data collection, either exclusively^43 45 47 51^ or as part of a wider suite of qualitative data collection methods, including focus group methodology.^46^ Two studies used ethnographic approaches and included participant observation in their data collection.^44 46^ Three studies collected and reviewed archival online data, including social media posts,^50^ emails sent via a listserv community^44^ and online forum posts.^48^

### Characteristics of peer support after miscarriage

*Appendix 3* presents details of each peer support ‘intervention’ following the TIDieR checklist.^37^ None of the included studies reported all eight characteristics outlined in the checklist. Five studies reported remote provision of peer support delivered online in various formats; social media support groups (n=3),^43 45 50^ online forums (n=1),^48^ Listserv email community (n=1).^44^ All other peer support (n=4) was delivered face-to-face in group settings.^46 47 49 51^ Finally, only one of the included studies reported tailoring the support offer to address the specific needs of the population.^46^

### Quality assessment

As detailed in the table (online supplemental appendix 4), five of the included studies were rated as high quality,^43 44 46 47 50^ while the remaining four were rated as moderate.^45 48 49 51^ No studies were excluded based on quality rating. However, it is important to note that five studies lacked adequate consideration of the researcher–participant relationship and had little to no reflection on the researcher’s role within the study.^43 45 48 49 51^

### Thematic synthesis

Thematic synthesis resulted in three overarching themes and seven subthemes (table 2). ‘Engaging in relational recognition’ reflects the validation and connection that arises through experiential resonance, often heightened by the context of exclusion from broader social or clinical support. ‘Mechanisms of communality’ describe how communality is enacted through the dynamic of peer interactions, including modelling and facilitating grief, benchmarking physical change and mattering through reciprocity, highlighting mutual support and shared coping. ‘Dynamics of access’ consider factors which shape engagement, including changing needs of individuals across time and modalities of support and their effects. Table 3 reflects how each study contributed to the development of the themes and subthemes. Themes and subthemes are described below with first order (participant quotations) and second order (primary study author interpretations) verbatim quotations.

**Table 2:** Overarching themes and subthemes derived from thematic synthesis

	Overarching theme	Subtheme
1.	Engaging in relational recognition	1.1 Experiential resonance
		1.2 The context of exclusion
2	Mechanisms of communality	2.1 Modelling and facilitating grief
		2.2 Benchmarking physical change
		2.3 Mattering through reciprocity
3.	Dynamics of access	3.1 Changing needs of individuals across time
		3.2 Modalities of support and their effects

**Table 3:** Overarching themes and subthemes with study contributions

First author	1.1	1.2	2.1	2.2	2.3	3.1	3.2
Barta 2023^43^	X	X	X	X	X	X	X
Callen 2024	X	X	X	X	X	X	X
Capitulo 2002^44^	X	X	X	X	X	X	X
Conroy 2023^45^	X	X	X	X		X	X
Drake 2010^46^	X	X	X		X	X	X
Endo 2024^49^	X		X				
Jackson 2024^47^	X		X		X		
Kuchinskaya 2018^48^	X			X		X	
McCreight 2024^51^	X	X	X		X	X	X

### Theme 1: engaging in relational recognition: ‘people can have sympathy, but it just wasn’t the same’

This theme reflects the relational process of peer support, in which individuals acknowledge, validate and respond to one another’s lived experience through a distinctive framework of understanding. While participants may not have identical stories, the framework of pregnancy loss allowed participants to interpret their experiences, grounded in emotional and social complexities that often accompany miscarriage. This experiential resonance, as a subtheme, reflects how shared experiences enabled peers to understand and empathise with one another, fostering emotional connection and empathy. It is important, however, to understand relational recognition within the context of exclusion. This second subtheme recognises how pregnancy loss can be minimised, stigmatised and misunderstood in wider social settings. Intersecting identities can shape how experiences are recognised and validated as well as broader systemic and social marginalisation, whereby non-peers (ie, those who have not been through the experience of miscarriage) are perceived to not understand.

#### Subtheme 1.1: experiential resonance

Participants felt that peer support offered a unique connection with others through a shared sense of authentic and intuitive understanding. Lived experience enabled participants to connect with others’ stories, recognising aspects of themselves in those narratives, while others, in turn, recognised elements of the participants’ experiences within their own.

*…*the only way that I felt comforted was if it was coming from somebody else who knew that pain. Because, I mean, people can have sympathy, but it just wasn’t the same coming from anyone else. But like the minute somebody told me, like, “I’ve also had a miscarriage. I lost a baby,” it gave a feeling of comfort that I couldn’t get anywhere else. (1st order, Conroy 2023)

As such, peer support helped others feel that they were not alone. Participants spoke of the peer support space as ‘safe’, facilitated by empathic, non-judgemental support for those experiencing distress.

The sentiment of “*I know how you feel*” was commonly expressed or alluded to, reflecting a shared desire for emotional connection, where the opportunity to open up and sit ‘alongside’ distress was seen as a key facilitator of meaningful support.

just having everybody in the group as a sounding board to validate my feelings and know that, yeah, most of the feelings I’m having or have had, other people have had as well, and it’s just nice to be able to share that and to have that kind of common experience. (1st order, Drake 2010)

Peer support can foster emotional processing through collaborative questioning and reflection. Individuals used perspective-sharing to make sense of difficult experiences and co-create meaning, deepening connection and understanding.

You know, having that peer person, that support person…if it’s somebody who has gone through it, it might even be more beneficial because you go into this thing so blind like, “Okay, well, is what I’m feeling more…is it normal to be angry? Is it normal to cry? Like, why am I laughing? And then crying and then like hysterically laughing again? (1st order, Conroy 2023)

Through resonance and recognition, participants felt that peer support allowed acceptance and validation of experiences. The idea of finding, being accepted by, and being able to help similar others facilitated a sense of belonging to a supportive community.

…being able to share, being able to help, being able to relate to somebody else and somebody who’s maybe at an earlier stage of what you’re going through…it’s a safe place to talk about it. (1st order, Drake 2010)

While experiential resonance was a powerful facilitator of connection for many, its impact was shaped by the degree to which participants felt their identities and backgrounds were reflected and understood within the group.

I think it just made me feel better knowing that, girls young like me it’s happening to too. […] Like I didn’t know it was as frequent in younger girls. (1st order, Barta 2023)

#### Subtheme 1.2: the context of exclusion

Relational recognition in peer support is not only about connecting with someone with shared experience, but also about counteracting broader exclusion, whether structural, cultural or social. The context of exclusion subtheme importantly recognised that social categories such as race, gender and ethnicity interact to produce unique experiences of discrimination and marginalisation. While the shared lived experience of miscarriage fosters resonance, other intersecting identities can, for some, take on greater personal significance. This subtheme explores how cultural and social categories combined to shape exclusion and create barriers to support, both within peer support contexts and in wider society.

Men’s experiences of grief after miscarriage illustrate this intersectional dynamic. Two studies discussed men’s support experiences. One study focused specifically on partners’ experiences after miscarriage,^51^ whereas another made anecdotal reference to partners’ perspectives, often framed through the accounts of mothers.^44^ Societal expectations, gender roles and cultural norms were seen to influence how men navigated loss.

I think the men are excluded from the grieving process from the beginning and therefore never get around to using support because they are told from the beginning that they shouldn't need and don't deserve support. (1st order, Capitulo 2002)

Hegemonic constructions of masculinity contribute to stigma when men perceived demonstrating emotion as failing to embody the stoicism or emotional restraint expected of their gender.

It was hard because all the time when you’re growing up you’re told ‘you’re a big boy now’, when you fell in the playground you were told ‘big boys don’t cry, boys are strong’, you’re always fighting those emotions really, but when you lose your baby… (1st order, McCreight 2004)

Cultural differences were also noted as affecting men’s capacity or willingness to express grief.

Kira said that her husband and other fathers, did not get validation of their losses in the American culture. (1^st^ order, Capitulo 2002)

Within this context, men were ‘expected’ to be less emotionally affected than women, leading them to prioritise their partner’s grief over their own. The perception that men have only a supportive role neglects the meaning that they attach to their loss.

My husband broke down at the hospital while trying to deal with all the funeral home paperwork. A nurse told him to stop that now because he needed to be strong for his wife (1st order, Capitulo 2002)

In addition to gender, other social identities were linked to unique support needs. Age, race and ethnicity were all seen to influence grief experiences, with some participants from minoritised backgrounds feeling more comfortable discussing their loss with peers who shared similar racial or ethnic identity, rather than relying solely on the common experience of miscarriage.

I was wondering, how do Black women keep going? So I was really drawn to see, like, how we keep going. You say you had a miscarriage a month ago, but you’re back out doing everything, so. I was wondering, like, how do you Black women do it?” In this example, P5 also alludes to cultural expectations around pain and perseverance that are informed by race/ethnicity. (1st/2nd order, Barta 2023)

Conversely, comparison presented a significant challenge in these spaces, as participants sometimes engaged in comparative or competitive grief, downplaying their own loss when others were perceived to have experienced something worse.

some participants, like P13 [participant number 13], found it difficult to accept support from individuals perceived to be worse off than themselves; Participants characterized groups perceived as fostering competitive grief or “war stories” were similarly thought to be as unsupportive. (2nd order, Barta 2023)

Reflecting systemic and social marginalisation, participants expressed encountering people that did not recognise the loss.

A lot of people don’t recognize it as a baby…[to them] it’s an early loss, there must not have been much of a connection. (1^st^ order, Drake 2010)

A general lack of understanding about miscarriage and the associated grief within the broader community often led those most in need of support to feel invalidated in their experience. As such, participants felt that interacting with those with similar experiences brought a sense of connection that could not be found in wider society.

I can talk about that baby openly and not feel like I’m getting a roll of the eyes or ‘Why is she still hung up on that?’ (1^st^ order, Drake 2010)

the reason women feel the need to be a part of an [online] group is because society is not comfortable with parental grief after the loss of a child, especially an infant (1st order, Capitulo 2002)

Societal stigma was seen as a barrier to openly discussing miscarriage beyond close peer groups, often leading to the use of language that participants felt trivialised and minimised their experience of loss.

…cliché’s, which I do think are well-intended on the part of the person stating them, tend to minimize the loss experience and magnify the need for women to receive support over time. (1st order, Drake 2010)

This was noted particularly by male participants, who felt their loss was not only devalued, but their grief experience was too.

… ‘well meaning’ remarks from the wider community, for example, ‘never mind, you can always have another baby’, led to feelings of anger and despair. (2^nd^ order, McCreight 2004)

As a result, participants reported feeling isolated outside of the peer support group and were more likely to withhold experience-sharing outside of the group for fear of pity and invalidation, highlighting the significance of peer spaces, where recognition is possible.

Oh yeah, there are settings outside for the group where you have to guard what you say. You share too much and you feel like people are looking at you like you’re a bad mother, or people are looking at you like you’re a crazy mother, or not a mother. (1^st^ order, Drake 2010)

### Theme 2: mechanisms of communality: ‘*knowing that we are all not alone is a great feeling’*

Communality in peer support refers to the shared emotional connection that arose from lived experience, expressed through a community of empathic understanding, reciprocal story sharing and collective coping.^54^ The Mechanisms of Communality were dynamic; created and sustained through peer interactions. In this way, communality functions as a collective community resource that underpinned resilience and supported processing and adaptation after miscarriage, outlined across three subthemes: modelling and facilitating grief, benchmarking physical change and mattering through reciprocity.

#### Subtheme 2.1: modelling and facilitating grief

This subtheme reflected the role peers play in miscarriage peer support by enabling expression of grief through non-judgemental spaces for sharing and processing emotions, including the enduring nature of grief following miscarriage.

Going to the support groups gives hope, because I’m talking to people that are going through it, or have been through it. And it’s just like a really different level of comfort. (1st order, Conroy 2023)

Peer interactions were perceived to enable the expression of grief by affirming individuals’ ‘right to grieve’. Peers acted as compassionate holders of grief, supporting others through the processing of emotions by modelling progress.

*Things were so difficult that I didn’t know what to do before that. I just cried and cried when I attended memorial services for miscarried children.… All I did was say ‘I’m sorry’ repeatedly to my child. But I no longer go to memorial services for miscarried children, and I think I’m all right. Since I discovered that there was a place for my child, I now know that my child is always with me. (1st order, Endo 2024)*

The experiential knowledge of peers, understanding of what may help and anticipating what others might need, was considered valuable in facilitating coping and adaptation.

A sad welcome to all new members and please just take it one day at a time. It is okay to miss our angels every day, as long as we get past the being angry part. That is what I had the hardest time with. This group helps us vent and get past the anger, knowing we are all not alone is a great feeling. (1st order, Capitulo 2002)

The ability to share experiences supported participants’ grief journeys, transitioning past feelings of blame following loss; from guilt to self-compassion.

Listening to a peer process their own feelings associated with EPL [early pregnancy loss] often led to participants reminding the peer that they were not at fault for the outcome of their pregnancy. This in turn helped participants overcome their own misplaced feelings of guilt and self-blame, fostering self-kindness and self-understanding instead. (2^nd^ order, Conroy 2023)

Through this process, peers supported one another in the acceptance and adjustment to a “new normal” (1st order, Drake 2010). Participants reflected on the idea of perpetual loss, suggesting that the impact of miscarriage is lasting and that the loss can make life feel profoundly different. One participant noted that having a baby does not erase the grief of a previous pregnancy loss.

None of us gets *over it* we leam to live with it, but we become and stay different people. (1^st^ order, Capitulo 2002)

Social comparison was sometimes viewed as a barrier to grief processing. Some participants perceived others as experiencing more severe circumstances and consequently felt less entitled to access the support they themselves required, a process consistent with downward social comparison. Although efforts were made to minimise such comparisons, participants noted that avoiding them entirely was challenging, and in some cases, these comparisons were seen as an inherent part of processing the emotional impact of miscarriage.

P13, for example, reflected that, “It just didn’t feel like I should be so upset, because [other women] had gone through such a more difficult experience.” (1^st^ order, Barta 2023)

In moving through the grief process, participants in six studies spoke about remembrance.^4344 46 49^,^51^ For many who had experienced miscarriage, being recognised as a mother held deep significance, even in the absence of living children.

feels a sense of recognition, both for her grief and for the very reality that she is, in fact, a mother. (2^nd^ order, Drake 2010)

Recognition of their loss as a baby/child by the wider community was equally important to their identity.

“The women in the group are recognizing the significance of the loss, and not only that, but they are viewing the child lost to miscarriage, in particular, as a child. They see that child as a baby and as a person, nothing less. (2^nd^ order, Drake 2010)

As such, participants supported one another with ideas of how they memorialised their losses. Practices such as lighting candles, engaging in prayer, displaying photographs and seeking symbolic representations, such as angels or butterflies, were described as meaningful acts of remembrance. These rituals were perceived to provide comfort and foster a sense of ongoing connection with the baby, supporting individuals to navigate grief where external or societal support mechanisms were typically absent.

Since angels have appeared & communicated to just about every culture & religion, calling my child an 'angel’ also gives me hope that they might send me signs or communicate somehow, before I join them in the afterlife. What other word can convey those ideas? I think 'angel’ suits perfectly!” (1st order, Capitulo 2002)

Spiritual support also emerged as a recurrent theme within these memorialisation practices. For some participants, religious faith played a central role in coping with loss. It is notable, however, that much of the literature exploring the role of spirituality in bereavement is based on studies conducted in the USA, where religious affiliation and expression may be more culturally prominent. This context should be considered when interpreting these findings, as the significance and visibility of spiritual support may differ in societies with varying religious and cultural approaches to grief.

I think that would have been something important to me, that the other women kind of shared those same types of beliefs, you know, like a belief in heaven, a belief that there’s purpose for pain. I’m not going through this awful thing for no reason, that maybe there’s a greater purpose. (f1st order, Barta 2023)

However, while spiritual support was helpful for some, for others, it acted as a barrier, creating discomfort and disengagement from peer spaces.

Two women reported feelings of “discomfort” at times, based on their reported lack of biblical knowledge in comparison to others in the group or the fact that their faith community did not encourage the reading of the bible for oneself. (2nd order, Drake 2010)

#### Subtheme 2.2: benchmarking physical change

There was a desire from participants to understand the physical effects of miscarriage, including physical symptoms experienced during and after the event, as well as the need to process the loss and its impact on the body. Peers facilitated a unique support opportunity through the provision of informational support based on experiential first-hand knowledge; something that clinicians or other healthcare professionals cannot provide.

Yeah, because it was women who…knew exactly what I was going through, you know…even like getting the D&C that I needed was traumatic in and of itself as well. And so being able to—well, being able to talk to [people who had also experienced miscarriage] helped me decide what the next step was for what I was gonna do to try to remove the dead tissue, you know. Whether it was gonna be like a natural process, or the pills, or the D&C. So it was helpful, because they had already gone through it, you know. (1^st^ order, Conroy 2023)

I feel empty and lonely. One week ago, I was pregnant, and now I am just empty and depressed. (1^st^ order, Capitulo 2002)

Experiential knowledge was a common source of information and reassurance, with peers often serving as efficient validators of physical health concerns and frequently adopting the role of ‘lay medics’.

And so having a support person that’s been there to be like, “Oh, yeah, that happened to me too,” or “Oh, yeah, I don’t know if that is normal, and I would get that checked out.” You know, something like that. I kind of overreacted about what was happening and caused more stress on myself than I needed to have. (1st order, Conroy 2023)

While many requests for information focused on sharing experiential knowledge, some informational support offered was based on facts, with reference made to academic articles and evidence. Online forums were seen as a space for knowledge production, with individuals learning from others’ experiences, in addition to evidence-based information sharing.

Collective learning was generated by assembling a variety of experiences and reflecting on those experiences, as well as common health care practices, perspectives of doctors, and external sources of information, including references to academic research. (2^nd^ order, Kuchinskaya, 2018)

Over time, some participants shifted their focus towards trying again. Benchmarking against others’ success stories during this phase was seen to instil hope and confidence among group members.

That this friend had also been able to have children following loss furthered the appraisal support available via this comparison, perhaps especially as P13 was pregnant at the time of the interview. (2^nd^ order, Barta 2023)

#### Subtheme 2.3: mattering through reciprocity

Reciprocity in peer support referred to how both the person giving and the person receiving support gains from the interaction. Several studies highlighted how this was achieved and the unique mutuality of support which facilitated this. Such acts may benefit both those who receive and those who offer support through developing a sense of mattering. Mattering in peer support refers to the subjective experience of feeling valued, seen, and important to others within the support relationship or group.^55^ It encompasses the belief that one *matters to others* (is appreciated, respected, and cared for) and that one’s *contributions matter* (can make a meaningful difference to others). In peer support settings, mattering facilitated and enhanced connection, self-worth and mutual empowerment, reinforcing a sense of belonging and identity within the group.^56^

Some participants described a *“pay it forward” (2^nd^ order, Drake 2010*) orientation to peer support, in which receiving help fostered a desire to give back to others facing similar challenges.

Um, so at least, you know, volunteering, l feel like l can make like my own why and be able to help, or at least give some sort of reason because the lack of reason is miserable. lf l’m gonna go through it. l’d like to be able to at least help someone else who has to go through it.” (1st order, Jackson 2024)

This shift from receiving to providing support was often framed as a meaningful way to express gratitude for the care they had once received and shifting the significance out of the loss towards feeling significant themselves.

the opportunity to provide support to newly bereaved parents, was their way of turning the negative experience into a positive one, like making lemonade out of lemons (2nd order, Jackson 2024)

This exemplified a shift from novice (ie, gaining experiential knowledge) to expert (ie, provider of experiential expertise).

I almost feel like I’m at the point where I would be maybe of more use helping someone else. If they needed someone to talk to or just listen to. (1^st^ order, Drake 2010)

There is also an opportunity, through shared connection, to foster friendships beyond peer support which is demonstrated in the findings of two of the included studies.^44 46^

These women in the group understood me and at the end of my pregnancy became my closest friends. (1st order, Capitulo 2002)

### Theme 3: dynamics of access: ‘*when you’re going through miscarriage, I think you need that support at that time’*

This theme explored how support needs are met in peer support spaces in both relevant and accessible ways. The subtheme changing needs of individuals across time reflected the dynamic interplay of temporality. The subtheme of modalities of support and their effects reflected the various forms of support offered and how support-seeking can be challenged or facilitated by the varying offers.

#### Subtheme 3.1: changing needs of individuals across time

Changing needs of individuals across time referred to the ways in which individuals experienced support and how their needs and expectations evolved. Within miscarriage peer support, grief is a highly individual process, and support needs vary across the period of engagement. Facilitation of these needs required understanding that loss is a dynamic process, rather than a fixed event, and that needs can be both time sensitive and ongoing.

I think just the wait times, the waitlists, for a lot of these services are really, really long, but when you’re going through your miscarriage, I think you need that support at that time. And it’s still nice to get it later on. But I think for me, the hardest month was when I was miscarrying. (1st order, Conroy 2023)

Individuals appeared to seek informational support in the period immediately following their miscarriage experience. This type of support appeared to help with coping on a practical level. Following this, there appeared to be a shift in support needs, with a move from informational support to emotional processing and sense-making.

In summary, most individuals wanted access to informational support at the time of their early pregnancy loss (EPL), while there was a pivot to a demand for emotional support following their EPL. (2nd order, Conroy 2023)

Although these shifts are generally experienced as positive and facilitative of the grief process, they were also seen to have adverse effects. In some cases, long-term engagement in peer support shifted from comfort to despair, as listening to and engaging with repeated loss narratives began to trigger the re-experiencing of earlier trauma. Such experiences were seen to hinder the processing of grief and made moving forward after miscarriage more challenging.

I did join a couple of Facebook support groups. I recently snoozed them, just because it’s a large group of women who are constantly posting about their losses and just what a hard time they’re having, so I just felt like a vortex of me just getting dragged down and not being supported. I just…I needed some distance. So you’re inundated with so much, especially since we’re hoping to try again soon, and I just don’t wanna be wrapped up in the anxiety of constant loss. (1st order, Conroy 2023)

Specific needs also emerged for those navigating pregnancy after loss, where heightened anxiety and fear often accompanied the experience.

We tried to get pregnant 2 months after we lost Jonah. Well, we got pregnant on the first try, and sure we were thrilled for about the first 5–10 min, then reality set in. The fear, the anxiety, the guilt, the anger, etc… all came rushing in full force and I was stressed! Well I am 15 weeks pregnant currently and I am not going to tell you that it is a breeze or it is easy… IT ISNT…I am a walking mess. (1^st^ order, Capitulo 2002)

Remaining in specific miscarriage groups while pregnant was seen to alter group dynamics and potentially inhibit the support seeking of others.

Although women report feeling happy for those in the group who are expecting, and I believe that they are, there is also some feelings of discomfort amongst the group. (2nd order, Drake 2010)

#### Subtheme 3.2: modalities of support and their effects

Across studies, different modalities of support were found to both facilitate and challenge support seeking. In-person groups were often valued for their role in coping with loss, yet practical barriers such as geography and cost were seen to be factors which may limit access.

However, the majority of participants wanted access to therapy or in-person support groups, but faced prohibitively long wait times, financial barriers, or logistical obstacles. (2^nd^ order, Conroy 2023)

In contexts such as the USA, where private healthcare predominates, therapy costs were noted as a prohibitive factor. Some participants perceived a lack of miscarriage specific knowledge in professional therapy compared with support from those with lived experience.

She [the therapist] said, “Yes, I have experience with grief and miscarriage.” But then anytime I brought up miscarriage in our conversations, it would get sidestepped or passed along. (1^st^ order, Conroy 2023)

Online support, through social media groups and forums, provided greater accessibility but introduced unique challenges. Some participants expressed apprehension about using social media for grief support.

I usually don’t get too into Facebook groups, butt…—it’s like “this is how it happened to me too” or even somebody just saying, “I’m sorry that you’re experiencing that,” or “You do have support; you’re not alone. (1^st^ order, Conroy 2023)

Even in moderated groups, exposure to distressing content and, at times, insulting comments could deter participation. Nonetheless, online forums were also described as offering distraction from otherwise triggering content and fostered a sense of connection.

Having so many people who are also… struggling with loss and going through the same experiences and worse, like, having their presence known makes it much more tolerable because then instead of just like scrolling and seeing baby after baby after baby, I just don’t feel as alone. (1^st^ order, Conroy 2023)

The ‘tolerance principle’ was used in one study^43^ to describe how individuals sometimes chose to remain in groups despite disempowering or negative aspects, managing their own discomfort through empathy for others’ needs.

Support was not limited to peer groups, with participants highlighting the role of wider networks, including friends, family, and even pets in providing comfort. Some also referred to personal coping strategies, such as distraction, as a means of managing grief.

Another mother said I cope as well as I can—I threw myself back into work just a few days after I miscarried with [baby’s name] and haven’t yet stopped long enough to let anything REALLY get me down” (1st order, Capitulo 2002)

## Discussion

This review provides a thematic synthesis of evidence about peer support for women, and their partners, after experiencing miscarriage. The findings address the review question by identifying both barriers and facilitators to miscarriage peer support by examining evidence from diverse studies. Nine studies were included in the review and thematic synthesis produced three overarching themes: ‘Engaging in relational recognition’, ‘Mechanisms of communality’ and ‘Dynamics of access’. Our findings underscore several general considerations for future implementation of peer support (*Appendix 5*). Additionally, discussed below, we consider how these barriers and facilitators relate to miscarriage-specific nuances which may be important considerations in future service design and provision of peer support, as well as suggesting practical actions that clinicians can take to support patients following miscarriage.

### Engaging in relational recognition

The unique benefits, and therefore key facilitators, of peer support after miscarriage are seen through empathy, affirmation and encouragement gained from relational interactions with peers. This is consistent with previous research in peer support which identifies talking/listening and socialising as key themes, highlighting the importance of creating and maintaining opportunities for peer connection and supportive dialogue.^24 54 57 58^

Active modelling of empathic and inclusive communication within peer support spaces facilitated connection by creating safe environments where miscarriage-related grief and parenthood could be actively acknowledged. While later pregnancy losses tend to be more publicly acknowledged, a support group may be the first opportunity that a peer can acknowledge their pregnancy, often providing the first space where people feel able to talk openly about their experience, without minimising or medicalising their loss. Challenges related to connecting with others within the wider community were widespread in our findings. In contexts where miscarriage remains highly stigmatised, private or even taboo, peer spaces uniquely validate loss experiences, offering recognition not typically addressed within general mental health support.^59^

Our findings also highlight the important and nuanced role peer support has in affirming parenthood after miscarriage, even if others never saw them as a parent. Previous research suggests that those who experience pregnancy loss may experience secondary losses, associated with but distinct from the loss of their child.^60^ The loss of possibility, future identity, routines and relationships that were imagined are all cited in previous literature as additional factors to grief after miscarriage.^60^,^63^ The lack of public acknowledgement of early pregnancy loss limited opportunities for open disclosure, leaving some individuals without a space to articulate their grief or parental identity outside of peer-support contexts. As such, miscarriage peer support groups play an important role in normalising a grief that, it is perceived, society often disenfranchises through a lack of recognition and understanding.^64 65^

A key facilitator of miscarriage peer support is the role that the peer support facilitator plays in minimising potential barriers related to inclusivity; taking into consideration broader cultural and societal contexts to meet the diverse needs of individuals. The flexibility to tailor peer support to individual and contextual circumstances acts as a key facilitator of support. Our study found that intersecting identities such as gender, race, culture and age influenced individuals’ willingness and ability to engage in support. Failure to account for individual and contextual differences acted as a barrier, constraining the tailoring of support and limiting its perceived relevance for some service users. For example, findings suggested that men and some racial or cultural groups may feel excluded in peer support settings and may prefer concordant spaces which reflect their needs and values. Previous research found men are less likely to seek help in the context of mental health and a recent review of men’s experience of pregnancy loss found that men felt that they needed to conform with societal expectations in relation to their behaviour and emotional expression.^66 67^

### Mechanisms of communality

This overarching theme overlies the structural conditions of togetherness that peer support brings, through collective identity and practice through peer interactions. This is consistent with previous research in peer support for mental health suggesting that peer support can provide a supportive community and reduce social isolation.^26^ Opportunity to model and facilitate grief experiences through sharing emotions and experiences allows grief to be held as a collective process rather than an individual burden.^68^

Role modelling within peer support groups facilitated hope, empowerment and coping, as participants were inspired by others’ resilience and capacity to navigate loss, supporting the development of their own coping strategies.^30 69 70^ Conversely, shared experience could also act as a barrier when social comparison led to discomfort, feelings of guilt or perceptions of a ‘lesser need’ for support. Congruent with other studies exploring ‘hierarchies of loss’, hierarchies of social recognition felt exclusionary to help-seeking for some participants.^60^ It is important that peer support facilitates non-hierarchical sharing of grief experiences. Reducing competitive grief ensures that those who have experienced miscarriage are met with the concept that *‘every experience is valid’*.^60^

Peer spaces can validate memorial rituals in ways that general mental health peer support typically would not. While later pregnancy losses are often memorialised with rituals such as funerals, obituaries and memory boxes, our study found that many participants who experienced miscarriage did not feel included in bereavement support in the same way. Previous bereavement studies have demonstrated the value of collective practice to allow grief to be held communally.^68 71^ Payne and colleagues highlight how memory-making can support bereaved women to maintain a sense of parental identity after perinatal loss.^72^ While peer-support spaces facilitated collective grief and validation of miscarriage through memorial and remembrance practices, the lack of comparable recognition in formal support settings acted as a barrier, contributing to feelings of exclusion and unacknowledged loss. Peer-led approaches were particularly important to address this gap through shared, experiential knowledge.

Miscarriage involves biological recovery which is often overlooked.^73^ Support groups facilitate the sharing of lived experience, providing service users with access to first-hand knowledge beyond that available in clinical spaces. Previous research also suggests the positive impact of sharing experiences of navigating systems or physical symptoms on mental well-being.^24 58^ Miscarriage often occurs at home and is commonly managed outside hospital settings, at home or via outpatient settings, without healthcare professional presence.^1^ Peer support is therefore often utilised to seek first-hand, body-specific guidance about procedures and post-loss symptoms that clinical encounters may not provide at the time of need. The physical trauma associated with miscarriage, in addition to the complex overlay of emotional grief that some experience, is often missed in general mental health support and potentially in mixed pregnancy loss settings. As such, our review suggests that miscarriage-specific groups, convened with peers who can share experiential knowledge, can be an important addition to psychosocial care available after miscarriage. Additionally, facilitating the provision of self-advocacy resources may be useful to support mothers in medical or family conversations and decision-making following their experience, as has been demonstrated in previous peer support research.^74 75^

Our review found that long-term members of miscarriage support groups tend to be increasingly motivated by their ability to help others. Research suggests that other peer support settings may remain more informational or therapeutic in nature. Miscarriage peer support is defined by its uniquely reciprocal and communal nature, where the act of ‘giving back’ is intertwined with validation and healing.^76^ The dynamic shift, from gaining experiential expertise to becoming an ‘expert by experience’, was found to be an important transitional process for some participants. Previous research suggests that this can only occur once individuals have processed their own grief sufficiently to enable them to support without re-traumatisation.^77^ In such cases, sharing becomes an act of solidarity and a way to transform personal loss into a resource for others.

### Dynamics of access

Accessible and timely peer support acted as a key facilitator by enabling a space for participants to process emotions in a time-sensitive and meaningful way. Congruent with other studies, our findings revealed a wide range of grief reactions were experienced after miscarriage.^78 79^ The emotional impact of miscarriage is unique; anticipatory grief centred around expected due dates and anniversaries of loss and anticipatory anxiety around future pregnancies exacerbate grief experiences.^60 61^ Peers can validate and normalise how ambiguous grief after miscarriage can resurface cyclically and often, unpredictably. Flexible access to peer support allows participants to opt into spaces which best meet their emotional needs at any given time. Clearly communicating the intent and tone of peer support spaces ahead of time further facilitated informed participation.

Conversely, fluctuating emotional states and uncertainty surrounding miscarriage experiences could act as a barrier to support. In comparison to later loss experiences, there is often less data or clarity about what happened during a miscarriage. As such, miscarriage, and particularly recurrent miscarriage, can lead to anxiety and deep fears about infertility and future loss.^80^ Pregnancy loss can spoil the expectation of parenthood and the experience of pregnancy as a happy or celebratory period.^60 61^ Instead, and congruent with previous studies, the review findings revealed that the experience of miscarriage often led to heightened anxiety in subsequent pregnancies.^61^ These factors complicated the sharing of experiences within peer spaces and could lead to emotional discomfort if expectations and sensitivities, such as disclosure or pregnancy after loss, were not clearly managed. Signposting to appropriate support for those who become pregnant after loss is important to allow support to evolve with changing needs.

Offering multiple modes of peer support engagement, including in-person, online and anonymous participation through forums and email, facilitated inclusive access by allowing participants to choose support that aligned with their preferences. Online and anonymous options, for example, enabled participation for those seeking anonymity. However, some modes of support, particularly online forums, also acted as barriers when exposure to distressing content or insulting comments negatively affected participants’ well-being. Previous studies have shown that long-term involvement in support and exposure to distressing content online can have negative impact on recovery.^81^ While offering multiple modes of engagement facilitated accessibility and choice, different support formats carried distinct risks and benefits and participants should be informed of the potential positive and negative impacts of different modalities to enable informed and autonomous decisions about engagement.

### Strengths and limitations of the thematic synthesis

The current review has several methodological strengths. The review protocol was registered in advance on PROSPERO to adhere to best practice guidelines for transparency. Forward citation searching strengthened the review by helping identify relevant and more recent studies that might not have been captured through database searches alone. Additionally, a second reviewer screened a proportion of studies at every stage to reduce potential biases.

Some limitations of the review must be considered. A key limitation of this review is the restriction to English-language studies, which may have introduced language bias and excluded relevant insights published in other languages. There is an ongoing debate about the value of quality ratings in reviews of qualitative studies. We used the CASP tool as a quality rating, rather than as an exclusion criterion.^40 82^ Sandelowski and Barrosso^82^ observe that qualitative studies tend not to provide enough methodological detail, but when the aim is to reflect on experiences, they consider that if the study quality is sufficient, it would be a mistake to exclude the rich, authentic descriptions they offer. As such, utilising quality assessment tools such as CASP may represent a limitation, as such frameworks often privilege methodological transparency over interpretative richness and therefore may risk undervaluing studies based on reporting detail.

### Considerations for practice

These findings highlight several practical actions that clinicians can take to support patients following miscarriage. Clinicians play a critical role in acknowledging miscarriage as a bereavement. Their role in validating a range of emotional responses and normalising the unpredictable nature of both miscarriage and grief is important. Pro-active signposting, including discussion of peer support options, may support informed engagement. Timely referral to appropriately designed, miscarriage-specific peer support resources, including flexible and non-clinical options, may further enhance support during different stages of recovery. Table 4 provides an overview of considerations for providers of peer support that can be derived from this study.

**Table 4:** Practice considerations

Overarching theme	Considerations
Engaging in relational recognition	Create and maintain opportunities for supportive dialogue and peer connection Use trauma-informed, loss-sensitive language Emphasise peer-led, non-hierarchical and validating structures that can be culturally adapted Acknowledge diversity upfront and consider cultural differences—normalise use of preferred languages, cultural references and traditions where appropriate
Mechanisms of communality	Centre peer spaces/interactions on ‘being heard’, creating a communal permission for grief—validate and normalise emotions and reactions Avoid highlighting comparison—‘every experience is valid’ Symbolic acts of remembrance can support a more collective process of grief Share (and archive with consent) personal stories of experiences (eg, physical symptoms, navigating systems) Provide resources for self-advocacy in medical or family conversations Create pathways to development—peer-to-peer mentoring
Dynamics of access	Provide dedicated non-clinical spaces where there is no expectation to discuss grief Offer multiple formats with meetings scheduled at varying times to accommodate schedules Allow anonymous participation where appropriate Allow people to opt-in to spaces which best meet their emotional needs at any given time Use appropriate facilitation, including opt-out points and reflective breaks Build group agreements around sharing limits/sensitive pregnancy disclosures Clearly delineate the intent and tone of each session ahead of time to allow informed choice in participation Have clear privacy/confidentiality policies

Considerations for peer support providers are framed within NHS principles of trauma-informed and loss-sensitive care, emphasising safety, trust, empowerment and cultural responsiveness.

NHS, National Health Service.

### Limitations of the review

It is however important to highlight that findings are based on a limited pool of research. Studies included in this review were conducted across four countries, most of them in the USA. As such, varying healthcare systems may influence study results, for example, through private healthcare provision and different working models of peer support. The variation of design, setting, and definitions of peer support within the included studies may have contributed to heterogeneity in reported experiences. Several studies provided limited detail on intervention characteristics or implementation context. This variability restricts our ability to fully assess mechanisms of action or contextual influences and therefore limits the extent to which findings can be directly compared across studies. Moreover, most of the studies focused on peer support for women/birthing people; only one study focused on partner perspectives of support. This reflects previous research which recognises the difficulties in recruiting men to healthcare-related research,^83^ and particularly in reproductive research.^84^

These limitations acknowledge that the barriers and facilitators suggested as considerations for practice are not intended to be strictly definitive, rather, they represent suggestions grounded in lived experiences and informed by the authors’ interpretations of the extant literature.

### Future research

To expand knowledge of the peer support experience after miscarriage, future research should focus on exploring the tailoring of miscarriage specific peer support in more depth, particularly those relating to intersectionality and the specific support needs based on gender, race and culture. Longitudinal narrative studies could give greater insight into the changing perceptions and needs associated with grief after miscarriage. Such studies would tell us more about how peer support may shift to better support long-term self-management. Further evaluation to establish efficacy, in terms of recovery-orientated outcomes, and cost-effectiveness of peer support, is needed.

## Conclusions

This review addressed the review question through a thematic synthesis, demonstrating that miscarriage peer support is shaped by a dynamic interplay of barriers and facilitators that influence engagement, acceptability and perceived value. Although we found parallels with existing research of peer support in general mental health, our results extended the previous research by providing novel insight into the support needs of those who experience miscarriage. Key facilitators included the use of non-hierarchical, communal spaces where loss is recognised, and peers feel that they have permission to grieve. Sharing of lived experience, role modelling, flexible and timely access, and the availability of multiple modes of engagement were also important. Conversely, barriers emerged where support structures failed to account for contextual nuance, which may influence support seeking. Future research should consider implementation issues using co-design approaches. In conclusion, our data indicate the importance of incorporating nuanced miscarriage-specific considerations when designing, implementing and adopting peer support provision for those who have experienced miscarriage.

## Supplementary material

10.1136/bmjopen-2025-113671 online supplemental file 1

## Data Availability

No data are available.
