# Supplementary material for: Barriers and facilitators to implementation of peer support after miscarriage: a systematic review using thematic synthesis methods
Source: BMJ Open. 2026 Jun 3;16(6):e113671. doi: 10.1136/bmjopen-2025-113671 (PMC13239413; doi:10.1136/bmjopen-2025-113671)
Supplement: online supplemental file 1 [file bmjopen-16-6-s001.docx]

**Appendix 1. Search Strategies**

Ovid MEDLINE(R) ALL <1946 to June 5, 2025>

1 peer*.ti,ab,kw.

2 ((lay or layperson* or laypeople or "lay people") adj3 (support* or led or lead* or deliver* or run* or held or direct* or online or "on line" or forum*)).ti,ab,kw.

3 (Parent to parent* adj3 (support* or led or lead* or deliver* or run* or held or direct* or online or "on line" or forum*)).ti,ab,kw.

4 ((Friend* or befriend) adj3 (support* or led or lead* or deliver* or run* or held or direct* or online or "on line" or forum*)).ti,ab,kw.

5 (mutual* adj2 (aid* or support* or help*)).ti,ab,kw.

6 (online adj2 (aid* or support* or help*)).ti,ab,kw.

7 peer group/ or peer influence/

8 Self Help Groups/

9 Social networking/

10 Social support/

11 or/1-10

12 (miscarriage* or miscarry*).ti,ab,kw.

13 (pregnan* adj3 loss*).ti,ab,kw.

14 (fetal loss*).ti,ab,kw.

15 exp Abortion, Spontaneous/

16 exp Perinatal Death/

17 "spontaneous abortion".ti,ab,kw.

18 "perinatal death".ti,ab,kw.

19 or/12-18

20 11 and 19

21 limit 20 to english language

22 limit 21 to yr="1990 -Current"

**CINAHL**

S1 TI peer* OR AB peer*

S2 TI ( ((lay or layperson* or laypeople or "lay people") N3 (support* or led or lead* or deliver* or run* or held or direct* or online or "on line" or forum*)) ) OR AB ( ((lay or layperson* or laypeople or "lay people") N3 (support* or led or lead* or deliver* or run* or held or direct* or online or "on line" or forum*)) )

S3 TI ( (Parent to parent* N3 (support* or led or lead* or deliver* or run* or held or direct* or online or "on line" or forum*)) ) OR AB ( (Parent to parent* N3 (support* or led or lead* or deliver* or run* or held or direct* or online or "on line" or forum*)) )

S4 TI ( ((Friend* or befriend) N3 (support* or led or lead* or deliver* or run* or held or direct* or online or "on line" or forum*)) ) OR AB ( ((Friend* or befriend) N3 (support* or led or lead* or deliver* or run* or held or direct* or online or "on line" or forum*)) )

S5 TI ( (mutual* N2 (aid* or support* or help*)) ) OR AB ( (mutual* N2 (aid* or support* or help*)) )

S6 TI ( (online N2 (aid* or support* or help*)) ) OR AB ( (online N2 (aid* or support* or help*)) )

S7 (MH "Peer Group") OR (MH "Peer Counseling")

S8 (MH "Support Groups")

S9 (MH "Social Networks")

S10 (MH "Support, Social+")

S11 S1 OR S2 OR S3 OR S4 OR S5 OR S6 OR S7 OR S8 OR S9 OR S10

S12 TI ( (miscarriage* or miscarry*) ) OR AB ( (miscarriage* or miscarry*) )

S13 TI ((pregnan* N3 loss*) OR ("fetal loss") OR ("spontaneous abortion") OR ("perinatal death")) OR AB ((pregnan* N3 loss*) OR ("fetal loss") OR ("spontaneous abortion") OR ("perinatal death"))

S14 (MH "Abortion, Spontaneous+")

S15 (MH "Perinatal Death")

S16 S12 OR S13 OR S14 OR S15

S17 S11 AND S16

S18 S11 AND S16

S19 S11 AND S16

Narrow by Language: - english

Limiters - Publication Year: 1990-2024

**APA PsycINFO**

S1 TI peer* OR AB peer*

S2 TI ( ((lay or layperson* or laypeople or "lay people") N3 (support* or led or lead* or deliver* or run* or held or direct* or online or "on line" or forum*)) ) OR AB ( ((lay or layperson* or laypeople or "lay people") N3 (support* or led or lead* or deliver* or run* or held or direct* or online or "on line" or forum*)) )

S3 TI ( (Parent to parent* N3 (support* or led or lead* or deliver* or run* or held or direct* or online or "on line" or forum*)) ) OR AB ( (Parent to parent* N3 (support* or led or lead* or deliver* or run* or held or direct* or online or "on line" or forum*)) )

S4 TI ( ((Friend* or befriend) N3 (support* or led or lead* or deliver* or run* or held or direct* or online or "on line" or forum*)) ) OR AB ( ((Friend* or befriend) N3 (support* or led or lead* or deliver* or run* or held or direct* or online or "on line" or forum*)) )

S5 TI ( (mutual* N2 (aid* or support* or help*)) ) OR AB ( (mutual* N2 (aid* or support* or help*)) )

S6 TI ( (online N2 (aid* or support* or help*)) ) OR AB ( (online N2 (aid* or support* or help*)) )

S7 (DE "Peer Support") OR (DE "Peer Counseling")

S8 DE "Support Groups" OR DE "Online Support Groups"

S9 DE "Social Networks" OR DE "Online Social Networks"

S10 DE "Social Support"

S11 S1 OR S2 OR S3 OR S4 OR S5 OR S6 OR S7 OR S8 OR S9 OR S10

S12 TI ( (miscarriage* or miscarry*) ) OR AB ( (miscarriage* or miscarry*) )

S13 TI ((pregnan* N3 loss*) OR ("fetal loss") OR ("spontaneous abortion") OR ("perinatal death")) OR AB ((pregnan* N3 loss*) OR ("fetal loss") OR ("spontaneous abortion") OR ("perinatal death"))

S14 DE "Spontaneous Abortion"

S15 S12 OR S13 OR S14

S16 S11 AND S15

S17 S11 AND S15

S18 S11 AND S15

Limiters - Published: 19900101-20241231

Narrow by Language: - english

**Embase**

Embase <1974 to 2025 June 5>

1 peer*.ti,ab,kw. 178776

2 ((lay or layperson* or laypeople or "lay people") adj3 (support* or led or lead* or deliver* or run* or held or direct* or online or "on line" or forum*)).ti,ab,kw. 1638

3 (Parent to parent* adj3 (support* or led or lead* or deliver* or run* or held or direct* or online or "on line" or forum*)).ti,ab,kw. 117

4 (Parent to parent* adj3 (support* or led or lead* or deliver* or run* or held or direct* or online or "on line" or forum*)).ti,ab,kw. 117

5 ((Friend* or befriend) adj3 (support* or led or lead* or deliver* or run* or held or direct* or online or "on line" or forum*)).ti,ab,kw. 6629

6 (mutual* adj2 (aid* or support* or help*)).ti,ab,kw. 3292

7 (online adj2 (aid* or support* or help*)).ti,ab,kw. 4524

8 peer group/ 32247

9 self help/ 15136

10 social network/ or social support/ 144348

11 1 or 2 or 3 or 4 or 5 or 6 or 7 or 8 or 9 or 10 343156

12 (miscarriage* or miscarry*).ti,ab,kw. 33446

13 (pregnan* adj3 loss*).ti,ab,kw. 17801

14 fetal loss*.ti,ab,kw. 6523

15 "spontaneous abortion".ti,ab,kw. 12155

16 "perinatal death".ti,ab,kw. 4865

17 spontaneous abortion/ 53930

18 perinatal death/ 5341

19 12 or 13 or 14 or 15 or 16 or 17 or 18 86903

20 11 and 19 895

21 limit 20 to (english language and yr="1990 -Current") 848

**Cochrane CENTRAL**

ID Search Hits

#1 (peer*):ti,ab,kw

#2 (((lay or layperson* or laypeople or "lay people") near/3 (support* or led or lead* or deliver* or run* or held or direct* or online or "on line" or forum*))):ti,ab,kw

#3 ((Parent to parent* near/3 (support* or led or lead* or deliver* or run* or held or direct* or online or "on line" or forum*))):ti,ab,kw

#4 ((Friend* or befriend) near/3 (support* or led or lead* or deliver* or run* or held or direct* or online or "on line" or forum*))

#5 ((mutual* near/2 (aid* or support* or help*))):ti,ab,kw

#6 ((online near/2 (aid* or support* or help*))):ti,ab,kw

#7 MeSH descriptor: [Peer Group] this term only

#8 MeSH descriptor: [Peer Influence] this term only

#9 MeSH descriptor: [Self-Help Groups] this term only

#10 MeSH descriptor: [Social Networking] this term only

#11 MeSH descriptor: [Social Support] this term only

#12 (48-#11)

#13 ((miscarriage* or miscarry*)):ti,ab,kw

#14 ((pregnan* near/3 loss*)):ti,ab,kw

#15 (fetal NEXT loss*):ti,ab,kw

#16 MeSH descriptor: [Abortion, Spontaneous] this term only

#17 MeSH descriptor: [Perinatal Death] this term only

#18 ("spontaneous abortion"):ti,ab,kw

#19 ("perinatal death"):ti,ab,kw

#20 (11-#19)

#21 #12 AND #20

**Web of Science**

Peer* OR ((lay or layperson* or laypeople or "lay people") NEAR/3 (support* or led or lead* or deliver* or run* or held or direct* or online or "on line" or forum*)) OR (Parent to parent* NEAR/3 (support* or led or lead* or deliver* or run* or held or direct* or online or "on line" or forum*)) OR ((Friend* or befriend) NEAR/3 (support* or led or lead* or deliver* or run* or held or direct* or online or "on line" or forum*)) OR (mutual* NEAR/2 (aid* or support* or help*)) OR (online NEAR/2 (aid* or support* or help*)) OR "self-help group*" OR "social networking" OR "social support"

miscarriage* OR miscarry* OR pregnan* NEAR/3 loss* OR " fetal loss*" OR "spontaneous abortion" OR "perinatal death"

Limit to 1990, English language

**LENS.org**

( Title: ( miscarriage OR ( "pregnancy loss" OR ( "fetal loss" OR ( "spontaneous abortion" OR "perinatal death" ) ) ) ) OR ( Abstract: ( miscarriage OR ( "pregnancy loss" OR ( "fetal loss" OR ( "spontaneous abortion" OR "perinatal death" ) ) ) ) OR ( Keyword: ( miscarriage OR ( "pregnancy loss" OR ( "fetal loss" OR ( "spontaneous abortion" OR "perinatal death" ) ) ) ) OR Field of Study: ( miscarriage OR ( "pregnancy loss" OR ( "fetal loss" OR ( "spontaneous abortion" OR "perinatal death" ) ) ) ) ) ) ) AND ( Title: ( "peer support" OR ( "social support" OR ( "social networking" OR ( "social networks" OR "support groups" ) ) ) ) OR ( Abstract: ( "peer support" OR ( "social support" OR ( "social networking" OR ( "social networks" OR "support groups" ) ) ) ) OR Keyword: ( "peer support" OR ( "social support" OR ( "social networking" OR ( "social networks" OR "support groups" ) ) ) ) ) )

Limit to 1990-, journal article , preprint , dissertation

**British Nursing Index**

1. tiab(peer*) OR tiab(((lay OR layperson* OR laypeople OR "lay people") PRE/3 (support* OR led OR lead* OR deliver* OR run* OR held OR direct* OR online OR "on line" OR forum*))) OR tiab((Parent to parent* P3 (support* OR led OR lead* OR deliver* OR run* OR held OR direct* OR online OR "on line" OR forum*))) OR tiab(((Friend* OR befriend) P3 (support* OR led OR lead* OR deliver* OR run* OR held OR direct* OR online OR "on line" OR forum*))) OR tiab((mutual* P2 (aid* OR support* OR help*))) OR MAINSUBJECT.EXACT("Social support") OR MAINSUBJECT.EXACT("Social networks") OR MAINSUBJECT.EXACT("Support groups")
2. tiab(miscarriage* OR miscarry*) OR tiab(pregnan* P3 loss*) OR tiab("fetal loss*") OR tiab("spontaneous abortion") OR tiab("perinatal death") OR MAINSUBJECT.EXACT("Miscarriage")
3. Limit to English, 1990-

**HMIC Health Management Information Consortium <1979 to May 2024>**

1 peer*.ti,ab.

2 ((lay or layperson* or laypeople or "lay people") adj3 (support* or led or lead* or deliver* or run* or held or direct* or online or "on line" or forum*)).ti,ab.

3 (Parent to parent* adj3 (support* or led or lead* or deliver* or run* or held or direct* or online or "on line" or forum*)).ti,ab.

4 ((Friend* or befriend) adj3 (support* or led or lead* or deliver* or run* or held or direct* or online or "on line" or forum*)).ti,ab.

5 (mutual* adj2 (aid* or support* or help*)).ti,ab.

6 (online adj2 (aid* or support* or help*)).ti,ab.

7 exp Support groups/ or exp Peer groups/

8 exp Self help groups/

9 exp Social networking/

10 exp Social support/

11 or/1-10

12 (miscarriage* or miscarry*).ti,ab.

13 (pregnan* adj3 loss*).ti,ab.

14 fetal loss*.ti,ab.

15 "spontaneous abortion".ti,ab.

16 "perinatal death".ti,ab.

17 exp Miscarriages/

18 exp Perinatal mortality/

19 or/12-18

20 11 and 19

**ProQuest Dissertations and Theses**

1. tiab(peer*) OR tiab(((lay OR layperson* OR laypeople OR "lay people") PRE/3 (support* OR led OR lead* OR deliver* OR run* OR held OR direct* OR online OR "on line" OR forum*))) OR tiab((Parent to parent* P3 (support* OR led OR lead* OR deliver* OR run* OR held OR direct* OR online OR "on line" OR forum*))) OR tiab(((Friend* OR befriend) P3 (support* OR led OR lead* OR deliver* OR run* OR held OR direct* OR online OR "on line" OR forum*))) OR tiab((mutual* P2 (aid* OR support* OR help*)))
2. tiab(miscarriage* OR miscarry*) OR tiab(pregnan* P3 loss*) OR tiab("fetal loss*") OR tiab("spontaneous abortion") OR tiab("perinatal death")
3. Limit to English, 1990-

**Google Scholar targeted search**

allintitle: miscarriage peer OR "social support" OR "social networks" OR "social networking"

allintitle: miscarry peer OR "social support" OR "social networks" OR "social networking"

allintitle: "pregnancy loss" peer OR "social support" OR "social networks" OR "social networking"

allintitle: "fetal loss" peer OR "social support" OR "social networks" OR "social networking"

allintitle: "spontaneous abortion" peer OR "social support" OR "social networks" OR "social networking"

allintitle:"perinatal death" peer OR "social support" OR "social networks" OR "social networking"

Limited to 1990-

**Appendix 2. Characteristics of included studies**

| First Author  (year) | Country | Primary Aim and Focus | Participants  (Gender)  Age | Peer support format | Setting | Research design/ methods |
| --- | --- | --- | --- | --- | --- | --- |
| Barta (2023) | USA | To understand how comparison manifests in social media-based support groups for pregnancy loss—and with what affective consequences  Focus: Process, Outcome | 4 participants who specifically reported experiencing miscarriage  (Gender: Female n=3; Unspecified n=1)  Age: 27-35 years  Ethnicity:  Black/African American (n=1)  Hispanic (n=1)  Middle Eastern (n=1)  Black (n=1) | Social media support groups for pregnancy loss | Online | Qualitative study using semi-structured interviews with a cognitive mapping exercise |
|  |  |  |  |  |  |  |
| Callen (2024) | Australia | To understand how people impacted by miscarriage seek and are offered support online  Focus: Process | People who had experienced miscarriage  # of Ps unknown, analysis of 270 opening posts and 3484 responding comments  (Gender: not reported)  Age: not reported | Facebook miscarriage support group | Online | Qualitative study analysing archival social media posts |
| Capitulo (2002) | USA | To describe and interpret the culture of an online perinatal loss group  Focus: Process, Outcome | A total of 447 emails and 17 online surveys were analysed  (Gender: unable to determine)  Age: unable to determine | Listserv for miscarriage, stillbirth and neonatal death | Online | Ethnographic study (online) including archival review and qualitative open-ended survey feedback |
| Conroy (2023) | USA | To explore how patients emotionally cope with Early Pregnancy Loss (EPL) and to assess if there is interest in a peer EPL support program with a self-compassion component  Focus: Process, Outcome | 21 women who had experienced early pregnancy loss  (Gender: Female n=21)  Age: 18-50 years  Ethnicity:  Hispanic or Latino (n=3),  Not Hispanic or Latino (n=18)  Religion:  Religion  Atheist (n=1),  Protestant (n=4),  Jewish (n=1),  Catholic (n=6),  Buddhist (n =1)  No religion (n=14) | Facebook miscarriage support group: *“Empty Cradle”* | Online | Qualitative study using semi-structured interviews |
| Drake (2010) | USA | To explore the support experiences of bereaved mothers who participate in the ‘Mommies with Hope’ group  Focus: Process, Outcome | 7 women who have experienced miscarriage  (Gender: Female n=7)  Age: 27-34 years | Biblically based support group: *“Mommies with Hope”* | Face-to-face group support | Ethnographic study using qualitative interviews, focus group and participant observation |
| Endo (2024) | Japan | To understand what constitutes beneficial spiritual support for women who experience miscarriage or stillbirth and factors restoring mental strength and willpower  Focus: Process, Outcome | 16 women reported experiencing pregnancy loss before 22 weeks gestation  (Gender: Female n=16)  Age: unable to determine | Self-help group meetings | Face-to-face group support | Qualitative study using semi-structured interviews |
| Jackson (2024) | USA | To explore how being part of a peer support bereavement programme impacts women's journeys with perinatal loss  Focus: Process, Outcome | 7 women who reported experiencing miscarriage  (Gender: Female n=7)  Age:27-58 years | A group bereavement support programme:  *“Caring Companions”* | Face-to-face group support | Qualitative study using semi-structured interviews |
| Kuchinskaya (2018) | USA | To explore what kinds of knowledge are produced by online forum participants with Recurrent Pregnancy Loss (RPL) and how do they relate to the relevant medical knowledge and practices.  Focus: Process | Participants had experienced recurrent pregnancy loss (# of Ps unknown, analysis of 47 threads)  (Gender: not reported)  Age: unable to determine | Online forums | Online | Qualitative study analysing archival forum posts |
| McCreight (2024) | UK | To describe the experiences of men whose partners had experienced pregnancy loss, based on data from Northern Ireland  Focus: Process, Outcome | 8 men had experienced miscarriage  (Gender: Male n=8)  Age: not reported | Face-to-face support groups including Miscarriage Support Group | Face-to-face group support | Qualitative study using semi-structured interviews |

| Appendix 3. Summary of Studies with Peer Support Characteristics Based on TIDieR^1^ Checklist | | | | | | |  |  |
| --- | --- | --- | --- | --- | --- | --- | --- | --- |
| First Author  (year) | **Template for intervention description and replication (TIDieR)^1^** | | | | | | | |
|  | **Brief Name** | **Why** | **What** | **Who** | **How** | **Where** | **When** | **Tailoring** |
| Barta (2023) | Social media (unspecified names) | Share and read other’s experiences | Posts, storytelling, replies and threads | NR | Internet (various social media) | Online | Available to access 24/7 with Internet connection | NR |
| Callen (2024) | ‘Open’ Facebook group (unspecified name) | Make and respond to other’s posts | Posts, storytelling, replies and threads | Moderated by ‘administrators’ | Internet  Facebook group | Online | Available to access 24/7 with Internet connection | NR |
| Capitulo (2002) | listserv community  (unspecified name) | To connect participants with people in similar situations that otherwise wouldn't have been connected | Listserv (email list of people who have experienced perinatal loss) | Members of the Listserv.  List is created and maintained by a lead, but everyone part of the listserv can contribute freely and equally | Internet (email) | Online | Available to access 24/7 with Internet connection | NR |
| Conroy (2023) | *Empty Cradle* Facebook group | Make and respond to other’s posts | Posts, storytelling, replies and threads | NR | Internet Facebook group | Online | Available to access 24/7 with Internet connection | NR |
| Appendix 3. (Continued) | | | | | | | | |
| Drake (2010) | *Mommies with Hope* | A biblically-based support group for mothers who have experienced perinatal loss | Refreshments, bibles, passages from bibles (sometimes prepared), facilitation of conversation to highlight areas of concern, story telling | Two facilitators with lived experience of pregnancy loss | Face-to-face group delivery | Various meeting spaces including private rooms at a coffee shop, local church, meeting room at local church-owned building and community building | Once per month; 2-hours  No time restrictions on attendance | If someone does not have a personal bible, there will be spare available to follow passages, but this is not required.  Initial sharing of pregnancy loss story when new attendees.  Discussions tailored to individual concerns |
| Endo (2024) | Self-help groups (unspecified name) | NR | NR | NR | Face-to-face group delivery | NR | NR | NR |
| Jackson (2024) | *Caring Companions* | To help other grieving mothers and families navigate their loss journeys | Face-to-face support – discussions, story telling | Volunteers with lived experience of pregnancy loss | Face-to-face group delivery | NR | NR  No time restrictions on attendance | NR |
| Appendix 3. (Continued) | | | | | | | |  |
| Kuchinskaya (2018) | Online forums | To enable reflection and articulation of shared experiences of recurrent pregnancy loss (RPL) | Posts, storytelling, replies and threads | NR | Internet (various online forums) | Online | Available to access 24/7 with Internet connection | NR |
| McCreight (2024) | 3 x Self-help groups:  *Stillbirth and Neonatal Death Society*  *Remember Our Child*  *Miscarriage Support Group* | To provide support to men bereaved by pregnancy loss | Face-to-face support – discussions, story telling | Peer facilitators | Face-to-face group delivery | NR | Once per month | NR |

^1 Brief name: Name or phrase that describes the intervention; Why: goal of the intervention; What: procedures used in the intervention; Who: the expertise of who provided the intervention; How: the mode of delivery the intervention; Where: location and necessary infrastructure to the intervention (unable to determine); When: number of times the intervention was delivered and over what period, number of sessions and duration; Tailoring: If the intervention was planned to be personalized.^

**Appendix 4.** Critical appraisal of included studies using CASP checklist and scoring system

| First Author, Date | 1.  Aim | 2.  Method | 3.  Design | 4. Recruitment | 5. Data collection | 6.  Researcher role | 7.  Ethics | 8.  Analysis | 9.  Findings | 10.  Value | Overall  rating |
| --- | --- | --- | --- | --- | --- | --- | --- | --- | --- | --- | --- |
| Barta, 2023 | 1 | 1 | 1 | 1 | 1 | 0.5 | 1 | 1 | 1 | 1 | 9.5 |
| Callen, 2024 | 1 | 1 | 1 | 1 | 1 | 1 | 1 | 1 | 1 | 1 | 10 |
| Capitulo, 2002 | 1 | 1 | 1 | 1 | 1 | 1 | 1 | 1 | 1 | 1 | 10 |
| Conroy, 2023 | 1 | 1 | 0 | 1 | 1 | 0.5 | 1 | 1 | 1 | 1 | 8.5 |
| Drake, 2010 | 1 | 1 | 1 | 1 | 1 | 1 | 1 | 1 | 1 | 1 | 10 |
| Endo, 2024 | 1 | 1 | 0 | 1 | 1 | 0.5 | 1 | 0.5 | 1 | 1 | 8 |
| Jackson, 2024 | 1 | 1 | 1 | 1 | 1 | 1 | 1 | 1 | 1 | 1 | 10 |
| Kuchinskaya, 2018 | 1 | 1 | 0.5 | 0.5 | 1 | 0 | 1 | 0.5 | 0.5 | 1 | 8 |
| McCreight, 2004 | 1 | 1 | 1 | 1 | 0.5 | 0.5 | 0.5 | 0.5 | 1 | 1 | 8 |

*Note:* CASP scoring system: 1 = 1es, 0.5 = unsure, 0 = no. Scoring system: high (9-10), moderate (7.5-8.5), low (6.5-7), exclude scores below 6

| Appendix 5. Considerations for Future Implementation of Peer Support after Miscarriage | | | |
| --- | --- | --- | --- |
| Theme / Subtheme | **Barrier/ Facilitator** | **Key Insight** | **Practice Considerations** |
| Engaging in relational recognition  –  Experiential resonance | Facilitator | Empathy, affirmation, and encouragement gained from a supportive social space | Create and maintain opportunities for peer connection and supportive dialogue.  Facilitators should actively model empathetic communication, prioritising respect, validation and inclusivity. |
| Engaging in relational recognition  -  The context of exclusion | Barrier | Stigma or silence associated with non-peer interactions | Centre peer spaces/interactions on ‘being heard’ |
| Engaging in relational recognition –  The context of exclusion | Barrier | Societal expectations; mismatched experiences across loss type, race/culture, gender | Liaise with men/non-birthing partners to ascertain needs for support  Acknowledge diversity upfront and consider cultural differences  Use inclusive intake questions to match peer mentors and support partners where appropriate |
| Appendix 5. (continued) |  |  |  |
| Engaging in relational recognition –  The context of exclusion | Facilitator | Culturally responsive to diverse support needs | Emphasise peer-led, non-hierarchical, and validating structures that can be culturally adapted  Normalise the use of preferred languages, culture references and traditions |
| Mechanisms of communality  –  Modelling and facilitating grief | Facilitator | Validation and shared experience of grief | Built-in practices that create a communal permission for grief- validate and normalise emotions and reactions.  Symbolic acts of remembrance can support a more *collective* process of grief |
| Mechanisms of communality  –  Modelling and facilitating grief | Barrier | Divergent needs among participants  Social comparison; confronting others’ suffering | Clearly delineate the intent and tone of each session ahead of time to allow informed choice in participation  Avoid highlighting comparisons and train facilitators to guide non-hierarchical sharing of grief experiences – *“every experience is valid”* |
| Appendix 5. (Continued) |  |  |  |
| Mechanisms of communality  –  Benchmarking physical change | Facilitator | Access to first-hand knowledge unavailable elsewhere; informational support beyond clinical services | Share (and archive with consent) personal stories of experiences (e.g. physical symptoms, navigating systems)  Provide resources for self-advocacy in medical or family conversations |
| Mechanisms of communality –  Mattering through reciprocity | Facilitator | Learning while helping others; satisfaction gained from ‘giving back’ | Create pathways to development - peer-to-peer mentoring |
| Dynamics of access  –  Changing needs of individuals across time | Barrier | Re-traumatisation; competitive grief; group dynamics; evolving needs through stages of loss | Use appropriate facilitation, allow opt-out points and reflective breaks  Build group agreements around sharing limits/sensitive pregnancy disclosures – offering option for dedicated space for Pregnancy after Loss |
| Dynamics of access  –  Changing needs of individuals across time | Barrier | Emotional readiness may limit benefit | Dedicate non-clinical spaces with no pressure to talk about grief  Allow people to opt into spaces which best meet their emotional needs at any given time |
| Appendix 5. (Continued) |  |  |  |
| Dynamics of access  –  Modalities of support and their effects | Facilitator | Accessible delivery formats | Offer multiple formats with meetings scheduled at varying times to accommodate schedules  Use trauma-informed, loss-sensitive language |
| Dynamics of access  –  Modalities of support and their effects | Barrier | Accessibility and funding constraints | Offer multiple, free to access formats |
| Dynamics of access  –  Modalities of support and their effects | Barrier | Trust and privacy concerns in online settings | Have clear privacy/confidentiality policies  Allow anonymous participation where appropriate  Build trust gradually |
| Dynamics of access  –  Modalities of support and their effects | Facilitator | Flexible participation; support accessible for varying durations | Provide options to access support as and when required |
