# Supplementary material for: Barriers and facilitators to implementation of peer support after miscarriage: a systematic review using thematic synthesis methods
Source: BMJ Open. 2026 Jun 3;16(6):e113671. doi: 10.1136/bmjopen-2025-113671 (PMC13239413; doi:10.1136/bmjopen-2025-113671)
Supplement: Supplementary file 2 [file bmjopen-2025-113671.reviewer_comments.pdf]

PEER REVIEW HISTORY

BMJ Open publishes all reviews undertaken for accepted manuscripts. Reviewers are asked to complete a checklist review form and are provided with free text boxes to elaborate on their assessment. These free text comments are reproduced below.

ARTICLE DETAILS

Title (Provisional)

Barriers and facilitators to implementation of peer support after miscarriage: a systematic review using thematic synthesis methods

Authors

Burton, Leanne; Charles, Juanita; Cherry, M. Gemma; Corcoran, Rhiannon; Hassan, Shaima; Hill, Ruaraidh; Little, Sarah; Maden, Michelle; Mulholland, Helen; Perkins, Elizabeth; Slade, Pauline; Wallis, Selina; Marshall, Paul

---

VERSION 1 - REVIEW

**Reviewer**	**1**
**Name**	**Khan, Saima**
**Affiliation**	**Zayed University Abu Dhabi, College of Natural and Health Sciences**
**Date**	**16-Dec-2025**
**COI**	**None**

Thank you for the opportunity to review this manuscript. The paper addresses an important and timely topic and is generally well written and clearly structured. The research question is clearly defined, and the study design is appropriate to address the stated objectives. The methods are described in sufficient detail, and ethical considerations are appropriately addressed. The results are clearly presented and align well with the research question, and the discussion provides a balanced interpretation of the findings in relation to existing literature.

A few minor points may further strengthen the manuscript. The authors may consider clarifying certain methodological details to enhance transparency and replicability. Additionally, expanding the discussion of study limitations and their potential impact on the interpretation of findings would improve the overall rigor. Minor language and formatting refinements could also enhance clarity and readability.

Overall, this is a strong manuscript that makes a meaningful contribution to the field, and with minor revisions, it would be suitable for publication.

**Reviewer**	**2**
**Name**	**MacGregor, Becky**
**Affiliation**	**University of Birmingham, Applied Health Sciences**
**Date**	**13-Jan-2026**
**COI**	**None**

Thank you for an interesting and well written paper.

1. In your PROSPERO record you include two research questions, please consider including these in the manuscript or an explicit objective statement to fulfil the PRISMA criteria of explicitly stating the review objectives or questions. It is not clear from line 144 page 5 what this is.

2. In line 499 I think there is an 'as' missing

3. Please give consideration to structuring the discussion section based on the review questions rather than the results findings so that:

a) it is clear whether or not the review has answered the review questions

b) it is clear what the review has found are the barriers and facilitators for miscarriage peer-support.

4. In the Dynamics of access section of Table 4, I am unclear what 'dedicate non-clinical spaces with no pressure to talk about grief means'. Should it say dedicated? Please consider re-phrasing.

**Reviewer**	**3**
**Name**	**Joly, Rochelle**
**Affiliation**	**Weill Cornell Medicine, Obstetrics and Gynecology**
**Date**	**23-Apr-2026**
**COI**	**None**

The manuscript would be further strengthened by a brief, explicit statement outlining what clinicians can practically do to support patients following miscarriage in light of these findings. For example, highlighting the role of clinicians in acknowledging miscarriage as a bereavement, normalizing emotional responses, proactively discussing peer-support options, and providing timely referral or signposting to appropriately designed peer-support resources would help translate these insights into actionable clinical practice.

VERSION 1 - AUTHOR RESPONSE

Reviewer Comment	Author Response
**Reviewer 1 Comments**	
The authors may consider clarifying certain methodological details to enhance transparency and replicability.	Thank you for this helpful comment. In response, we have clarified several methodological details to enhance transparency and replicability. Specifically, we have clarified the literature search section (page 6, line 177-180) and the description of sample selection/eligibility criteria in the Methods section (pages 7, lines 193-203), including additional detail on definitions adopted for the review. We believe these revisions improve clarity and facilitate replication, while also tightening the methods section to avoid duplication of information.<br><br>Reporting follows the PRISMA guidelines, and additional methodological detail is provided in the Supplementary File (PRISMA checklist).
Additionally, expanding the discussion of study limitations and their potential impact on the interpretation of findings would improve the overall rigor.	We thank the reviewer for this valuable suggestion. In response, we have expanded the limitations section to provide a more explicit and detailed in consideration of the study's limitations and their potential impact on the interpretation of findings (pages 34-35, lines 860-873). Specifically, we now discuss data limitations and interpretation and how these factors may influence the conclusions drawn. We believe this strengthens the overall rigor and transparency of the manuscript.
Minor language and formatting refinements could also enhance clarity and readability.	We thank the reviewer for this suggestion. In response, we have made minor language and formatting refinements throughout the manuscript to improve clarity and readability. These edits do not alter the substantive content but aim to enhance the overall presentation.
**Reviewer 2 Comments**	
1. In your PROSPERO record you include two research questions, please consider including these in the manuscript or an explicit objective statement to fulfil the PRISMA criteria of explicitly stating the review objectives or questions. It is not clear from line 144 page 5 what this is.	We thank the reviewer for this comment. The PROSPERO record includes two research questions; however, the present manuscript addresses only the second of these. We recognise that this was not made sufficiently explicit in the original submission. To address this, we have revised the manuscript to include a clear objective statement specifying the research question addressed in this review (page 5, line 155). This amendment ensures compliance with PRISMA recommendations for explicitly stated review objectives.
2. In line 499 I think there is an 'as' missing.	Thank you for highlighting this typographical error; we have amended to include the missing 'as'.
3. Please give consideration to structuring the discussion section based on the review questions rather than the results findings so that:<br>a) it is clear whether or not the review has answered the	We thank the reviewer for this thoughtful suggestion. We chose to retain a results-led discussion structure, as this reflects the inductive nature of the thematic synthesis approach. While we have retained the existing structure, we have revised and strengthened the narrative to more explicitly reference the review question. This includes clearer signposting to indicate how the review question

review questions<br>b) it is clear what the review has found are the barriers and facilitators for miscarriage peer-support.	has been addressed, and more explicit synthesis of identified barriers and facilitators to miscarriage peer support; both within the discussion and conclusion. We believe these revisions improve clarity while maintaining the current structure of the Discussion.
4. In the Dynamics of access section of Table 4, I am unclear what 'dedicate non-clinical spaces with no pressure to talk about grief means'. Should it say dedicated? Please consider re-phrasing.	Thank you for your comment. We have re-phrased this statement accordingly and hope that this provides more clarity.
**Reviewer 3 Comments**	
The manuscript would be further strengthened by a brief, explicit statement outlining what clinicians can practically do to support patients following miscarriage in light of these findings. For example, highlighting the role of clinicians in acknowledging miscarriage as a bereavement, normalizing emotional responses, proactively discussing peer-support options, and providing timely referral or signposting to appropriately designed peer-support resources would help translate these insights into actionable clinical practice.	We thank the reviewer for this helpful suggestion. In response, we have added a brief, explicit statement outlining practical actions clinicians can take to support patients following miscarriage, informed by the review findings, to support Table 4 outlining practice considerations for those looking to deliver/provide peer support services.
